# Conserved regulatory modules and hub genes linking leaf transcriptomes to soybean seed weight

**DOI:** 10.1186/s12870-026-08603-w

**Published:** 2026-03-28

**Authors:** Jakob Bruggink, Julia Wozny, Ashkan Golshani, Elroy Cober, Bahram Samanfar

**Affiliations:** 1 Department of Biology, Carleton University, Ottawa, ON Canada; 2 Ottawa Research and Development Centre, Agriculture and Agri-Food Canada, Ottawa, ON Canada; 3 Department of Biology, University of Saskatchewan, Saskatoon, SK Canada

**Keywords:** Soybean, Seed weight, Co-expression networks, Differential expression, Transcriptomics

## Abstract

Soybean (*Glycine max* (L.) Merr.) seed weight is a critical yield component that varies significantly across Canadian growing regions due to environmental factors. Dissecting the genetic basis of this trait is challenging, as phenotypic plasticity often obscures stable regulatory mechanisms. This study aimed to identify stable gene co-expression networks associated with seed weight using multi-year transcriptomic data. We performed weighted gene co-expression network analysis (WGCNA) on RNA-seq data from leaf tissue collected from ten soybean genotypes grown across Eastern and Western Canada over four years (2018–2021). We identified five gene modules that were significantly correlated with seed weight and highly preserved across years. Functional analysis linked these modules to source-sink relationships, specifically circadian rhythm, photosynthesis, and lipid metabolism. Hub gene analysis identified known regulators *Reveille 1* and *Wrinkled 1*, validating the biological relevance of the networks. Furthermore, 18 of the top 50 hub genes co-localized with known quantitative trait loci (QTLs) for seed weight. Differential expression analysis revealed 11 hub genes with consistent regionally-linked expression patterns, suggesting potential targets for region-specific breeding. By focusing on network stability through module preservation analysis, we filtered out environmental noise to reveal core regulatory pathways connecting leaf gene expression during seed filling to seed weight at harvest. These stable hub genes represent high-confidence targets for breeding programs aiming to improve soybean yield stability across different geographies.

**Supplementary Information:**

The online version contains supplementary material available at 10.1186/s12870-026-08603-w.

## Background

Canadian farmers have increasingly adopted soybean (*Glycine max* (L.) Merr.) in crop rotations and this legume is now the third largest field crop in Canadian agriculture by farm cash receipts [1]. As global populations increase, Canadian soybean exports will continue to grow. Historically, Canadian soybean production has been predominantly concentrated in Eastern Canada, specifically Ontario and Quebec [2]. Compared to Western Canada, this region is characterized by a longer growing season, optimal temperatures, and consistent precipitation, leading to stable and high-yielding cultivation [2]. Recently, however, soybean acreage has rapidly expanded into Western Canada, including Manitoba and Saskatchewan [3]. This western expansion frontier presents distinct agronomic challenges, notably shorter growing seasons, cooler average temperatures, and altered photoperiods, necessitating the cultivation of early-maturing varieties (Maturity Groups 00 to 000). The stark contrast between the established eastern environments and the volatile western frontier provides an ideal natural framework for investigating how genotype x environment interactions dictate yield components such as seed weight, and for distinguishing between stable genetic networks and environmentally driven transcriptomic plasticity. In order to meet increasing demands, breeders work to expand this crop beyond its traditional growing regions while balancing yield drawdown due to environmental factors.

The yield of a harvest is determined by different factors including seed number and seed weight. Seed size is a more general term that refers to both the mass and volume of a seed and is therefore typically measured as 100-seed weight. In this text, seed weight is synonymous with 100-seed weight. It has previously been shown that soybean seed weight varies across Canadian growing regions [4, 5]. Understanding the genetic component of these differences is key to creating varieties that will thrive in Western Canada and similar regions around the world. This task is not simple since many confounding environmental variables affect seed weight, including but not limited to precipitation, sunlight hours, temperature, and soil type. Previous attempts to model the effects of these variables on agronomic traits have shown some success, such as a model developed in 2006 to determine environmental effects on yield and a more recent model combining environmental and genetic effects on soybean plant height from 2021; but, are limited by available data and may prove inaccurate as global weather patterns become more unpredictable [6, 7]. It is also important to note that expression patterns are impacted by both geographical differences and yearly weather differences. Therefore, an approach that can identify stable genetic factors despite this yearly environmental variability is required.

Weighted Gene Correlation Network Analysis (WGCNA) is a bioinformatic tool that can help elucidate stable expression patterns and associate them to traits of interest using weighted correlation calculations [8]. Using multi-year data and module preservation analysis allows for noise effects from inconsistent abiotic and biotic factors to be subdued, such as weather patterns and pathogen load. Combining these preserved networks with differential expression (DE) analysis allows for detection of geographically-dependent expression patterns associated with seed weight in soybean. The incorporation of traditional DE analysis after network construction provides a level of resolution not found in either tool individually. Understanding how geography influences co-expression networks is key to determining how growing region affects seed weight. The aim of this study was to implement an analysis technique that identifies consistent stable gene co-expression networks to provide a transcriptomic workflow for future investigations into the regulatory network associated with seed weight. We hypothesized that leaf transcriptional states during the R5 stage, the critical period for establishing carbon allocation capacity, act as upstream regulators of final seed weight.

## Methods

### Plant materials and experimental design

Ten soybean genotypes were grown across four locations in Canada from 2018 to 2021 [9]. These lines are as follows: X5583-1-041-5-5, AC Harmony, AAC Halli, 90A01, Maple Amber, OT13-08, OT14-03, AAC Springfield, Jari, and AC Proteus. They are publicly-available Canadian lines accessed through Germplasm Resources Information Network (GRIN). These lines were chosen for their varying seed protein content. The four locations represented eastern (Ottawa (latitude 45.39°, longitude − 75.72°)) and western (Morden (49.18°, − 98.08°), Brandon (49.86°, − 99.98°), Saskatoon (52.15°, − 106.57°)) growing regions. A single well-characterized Eastern site (Ottawa) served as the baseline environment, while three Western sites were utilized to capture the breadth of growing regions targeted by Canadian soybean breeders for future expansion. Leaf tissue was sampled at the R5 (early seed filling) growing stage for RNA extraction. Phenotypic data, including 100-seed weight, were collected as seen in Additional file 1. Climatic and soil data were also gathered and compiled in Additional file 2.

### RNA sequencing and data processing

Total RNA was extracted from approximately 200 mg of crushed leaf tissue using the SPLIT Total mRNA Extraction Kit (Lexogen, Vienna, Austria) following the manufacturer’s instructions. RNA quality was assessed using a NanoDrop™ 2000 Spectrophotometer (Thermo Fisher Scientific, Waltham, MA, USA), 1% agarose gel electrophoresis, and the TapeStation 4200 and 2100 Bioanalyzer systems (Agilent, Santa Clara, CA, USA). These quality control steps were performed at Génome Québec (Montréal, QC, Canada) and the Ottawa Research and Development Centre (Ottawa, ON, Canada). Samples exhibiting RNA integrity number (RIN) values of at least 5.0 and Q30 scores of at least 36 were selected for library preparation. To monitor sensitivity and quantification, the E0 Spike-in RNA Variant (SIRV) mix (Lexogen, Vienna, Austria), containing 69 isoform variants, was integrated into the RNA samples as an external control.

Paired-end sequencing was conducted using the Illumina HiSeq 4000 platform (Illumina, San Diego, CA, USA) at Génome Québec to generate cDNA libraries. The Canadian Centre for Computational Genomics processed the data using the GenPipes framework [10]. Quality control of the RNA-seq data was performed using dupRadar (v3.16, Biberach an der Riß, Germany; Bioconductor, R) to assess duplication rates, and edgeR (v3.16, Parkville, Victoria, Australia) was used for read normalization [11, 12]. Additional quality control steps included QualiMap (v2.2.1, Berlin, Germany) for feature alignment assessment and Preseq (v3.1.1, Los Angeles, CA, USA; Bioconductor, R) to estimate library complexity [13, 14]. RSeQC (v4.0.0, Nanjing, China; Bioconductor, R) was utilized for a comprehensive evaluation of read distribution, duplication, and junction saturation [15]. For data processing, Trimmomatic (v0.36, Jülich, Germany) was used to remove adaptor sequences and low-quality bases (phred score < 30) [16]. Trimmed reads were aligned to the Glycine max v2.1 genome (INSDC Assembly GCA_000004515.4) using STAR (v2.7.7a, Menlo Park, CA, USA) with the command --runMode alignReads [17]. Finally, read counts were generated using HTSeq (v0.12.3, Heidelberg, Germany) with the option -m intersection-nonempty [18].

Raw count files (available as Additional file 3) were processed for WGCNA. Low read counts were removed, retaining only genes with at least 5 counts in 2 or more samples. Data was separated by year and normalized using the Variance Stabilizing Transformation (VST) from the DESeq2 package in R [19]. Gene IDs that did not follow conventional ‘Glyma.’ format were removed, and the top 20% most variable genes (*n* = 9,461) were retained for network construction since genes lacking expression variation will create noise in the correlation detection algorithm [8].

### Phenotypic data analysis

Phenotypic data analysis was performed using the R statistical computing environment [20]. To evaluate the effect of growing region on 100-seed weight and seed oil content, parallel linear mixed-effects models (LMM) were fitted using the lme4 package [21]. The models included ‘Region’ (Eastern vs. Western Canada) as a fixed effect, while ‘Year’ and ‘Genotype’ were included as random intercept effects to account for temporal and genetic variance, respectively. Significance of the fixed effect was assessed using Satterthwaite’s method for approximating degrees of freedom, implemented in the lmerTest package [22]. Differences were considered statistically significant at *p* < 0.01.

### Co-expression network construction

Network construction was performed using the WGCNA R package [8]. To detect potential within-year technical batch effects and sample outliers, hierarchical clustering was performed on the VST-normalized expression data for each year prior to network construction. Because the WGCNA networks were constructed independently for each of the four years, cross-year sequencing batch biases were inherently avoided. A soft-thresholding power of 16 was chosen to achieve a scale-free topology fit of at least 0.75 across all years. The 2018 data’s function call was as follows *blockwiseModules(t(datExpr_clean$L18)*,* power = softPower*,* maxBlockSize = 30000*,* networkType = “signed”*,* TOMType = “signed”*,* deepSplit = 4*,* minModuleSize = 30*,* reassignThreshold = 0*,* mergeCutHeight = 0.2*,* numericLabels = TRUE*,* pamRespectsDendro = FALSE*,* saveTOMs = TRUE*,* saveTOMFileBase = “L18_TOM”*,* corType = “bicor”*,* maxPOutliers = 0.05*,* verbose = 5)*. The other three years had similar function calls only changing label parameters.

### Network analysis and module preservation

Module-trait associations were identified by calculating Gene Significance (GS) and correlating Module Eigengenes (MEs) with phenotypic traits using biweight midcorrelation (bicor). Modules were considered associated with seed weight if they exhibited an absolute correlation |r| ≥ 0.3 and an FDR-adjusted p-value < 0.01 in the 2018 reference dataset. Module preservation across the subsequent years (2019–2021) was calculated using the modulePreservation function with 200 permutations [23]. Modules were classified as highly preserved (Zsummary > 10), moderately preserved (2 < Zsummary < 10), or not preserved (Zsummary < 2). Only modules that were strongly associated with seed weight and highly preserved were retained for functional analysis. 

### Functional Annotation and Hub Gene Identification

Gene Ontology (GO) enrichment analysis was performed using SoyBase and significantly enriched (adj. p-value < 0.01) annotations were retained [24]. Hub genes were identified based on high module connectivity (kME ≥ 0.8) and strong trait correlation (|r| ≥ 0.5). The top ten hub genes per module were annotated using UniProtKB, TAIR10, Pfam, Panther, and KOG databases to determine biological function [25–29]. A QTL analysis was performed by cross-referencing hub gene genomic locations with known seed trait QTLs gathered from Soybase. Finally, regional differential expression of hub genes was assessed using a Wilcoxon rank-sum test with Benjamini–Hochberg FDR correction.

## Results

### Geographic variation significantly impacts soybean 100-seed weight

**Fig. 1:**
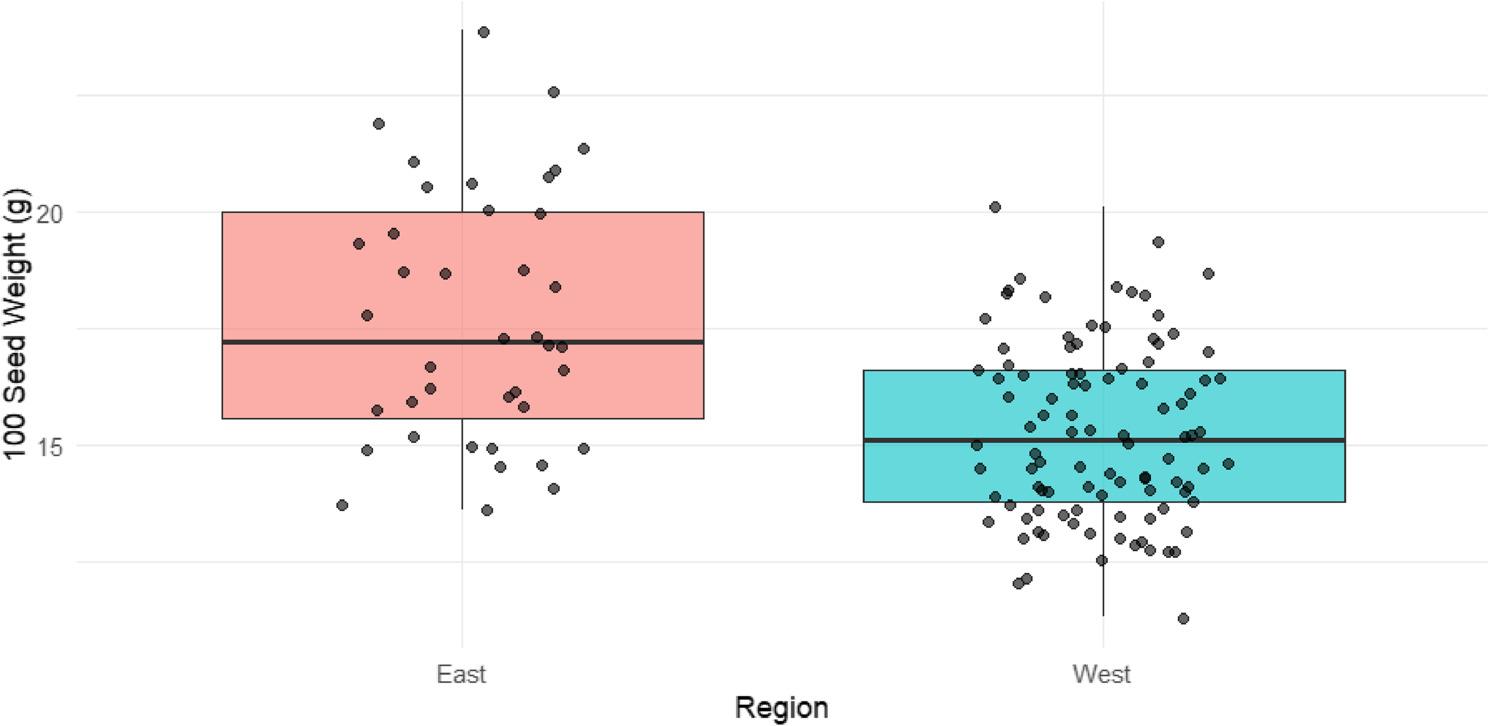
Regional differences in soybean 100-seed weight. Boxplots depicting the variance in 100-seed weight between Eastern and Western Canadian grown soybeans across four sampling years (2018–2021)

The 100-seed weight values collected for each sample can be found in Additional file 1. A linear mixed model was used to assess the effect of growing region on seed weight in R, accounting for random effects of year and genotype. 100-seed weight was significantly lower in Western Canada compared to Eastern Canada (regression coefficient = -2.45, t-statistic with 126 degrees of freedom = -6.83, *p* = 3.16e-10). The model estimates the mean 100-seed weight to be 17.7 g in the East and 15.3 g in the West and found them to be significantly different. This difference is visualized in Fig. 1 below. A parallel LMM was utilized to evaluate seed oil content across the regions. Mirroring the seed weight phenotype, total seed oil content was significantly lower in the Western environments compared to the Eastern samples (regression coefficient = -0.77, t-statistic with 126 degrees of freedom = -4.54, *p* = 1.29e-05). The model estimates a mean oil content of 20.73% in the East versus 19.96% in the West.

### Identification of highly preserved co-expression networks associated with seed size

**Fig. 2:**
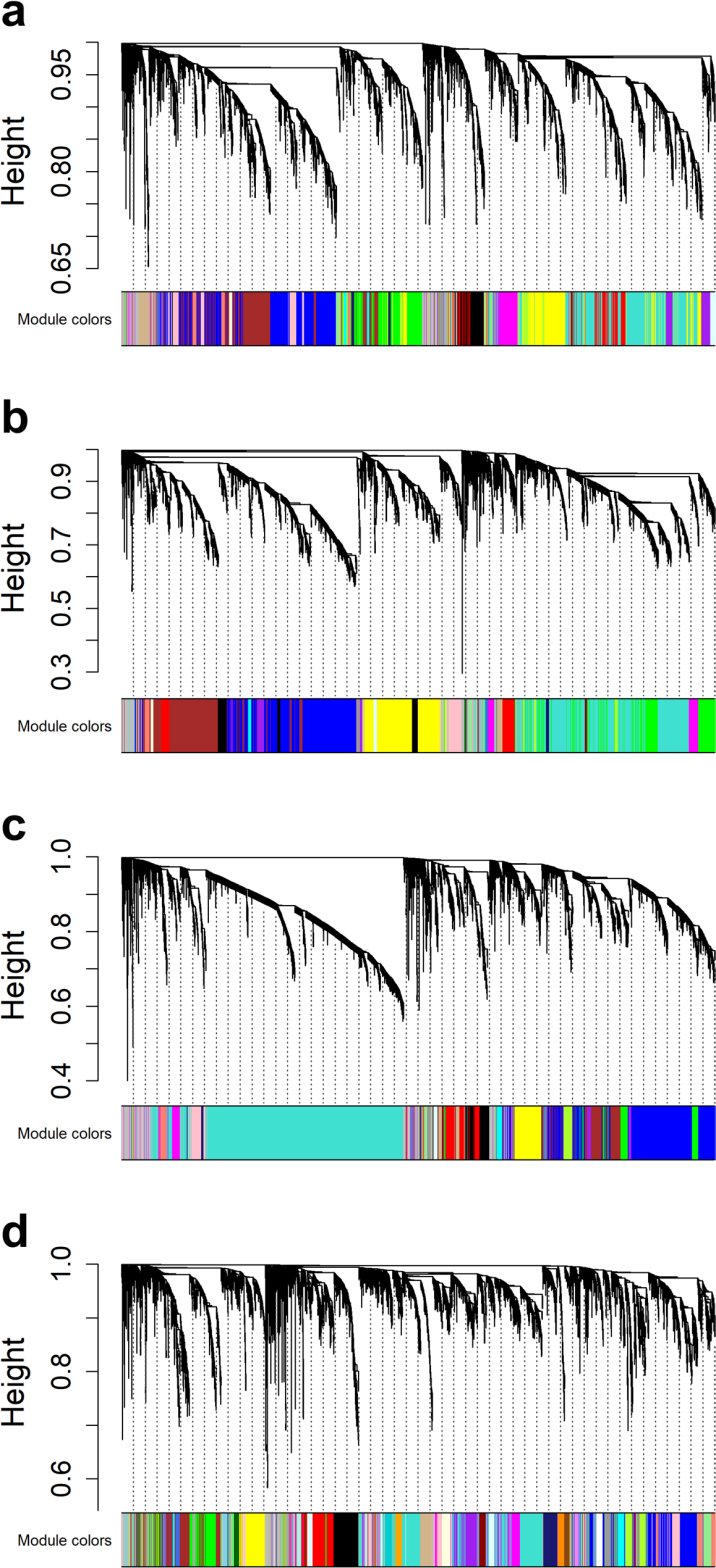
Module detection across four environments. Network dendrograms depicting module detection generated by the WGCNA R package for (**a**) 2018, **b** 2019, **c** 2020, and **d** 2021 expression data. Height is a measure of dissimilarity between gene expression patterns

Prior to network construction, hierarchical clustering of the VST-normalized samples within each individual year revealed no batch-driven clustering or significant outlier samples, confirming the technical reliability of the datasets. To improve interpretation across networks, consistent values were used in each call of the blockwiseModules() function from the WGCNA R package. A soft-thresholding power of 16 was found to provide a scale-free topology fit of at least 0.75 in all four years of expression data. The mean connectivity was 57 in 2018, 121 in 2019, 101 in 2020, and 27 in 2021. The other function variables were chosen to provide well-rounded modules that were not too large or too small as defined by (authors of package). The four networks were visualized using dendrograms as seen in Fig. 2.

**Fig. 3:**
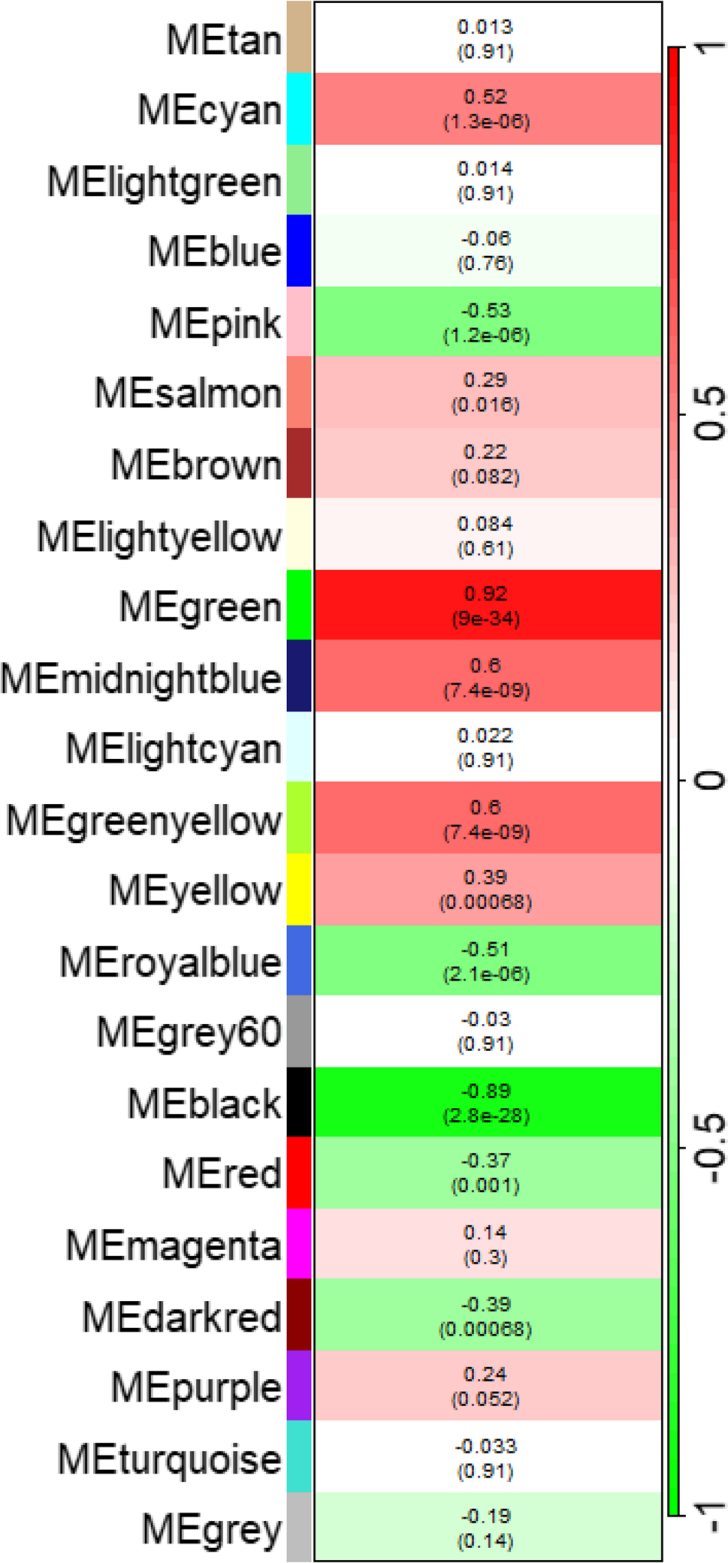
Module-trait associations for the 2018 reference network. Heatmap displaying the biweight midcorrelation (bicor) between module eigengenes (MEs) and 100-seed weight. Adjusted *p*-values are included in parentheses beneath the corresponding correlation value

Network modules were correlated to traits using MEs. MEs are the first principal component of the expression data and are used to summarize the expression of the genes in a module for a specific sample. Bicor was used to calculate the correlation between the MEs and the trait data to determine modules associated with agronomically important traits. Trait correlations can be seen in a heatmap in Fig. 3. Modules with an absolute correlation of at least 0.3 and an adjusted p-value of less than 0.01 for the 100-seed weight trait were chosen for further analysis. These modules were “black” (*r* = -0.89, *FDR-adjusted p-value = 2.8E-28*), “cyan” (0.52, *1.3E-6*), “darkred” (-0.39, *6.8E-4*), “green” (0.92, *0.9E-33*), “greenyellow” (0.6, *7.4E-9*), “midnightblue” (0.6, *7.4E-9*), “pink” (-0.53, *1.2E-6*), “red” (-0.37, *0.1E-2*), “royalblue” (-0.51, *2.1E-6*), and “yellow” (0.39, *6.8E-4*).

**Fig. 4:**
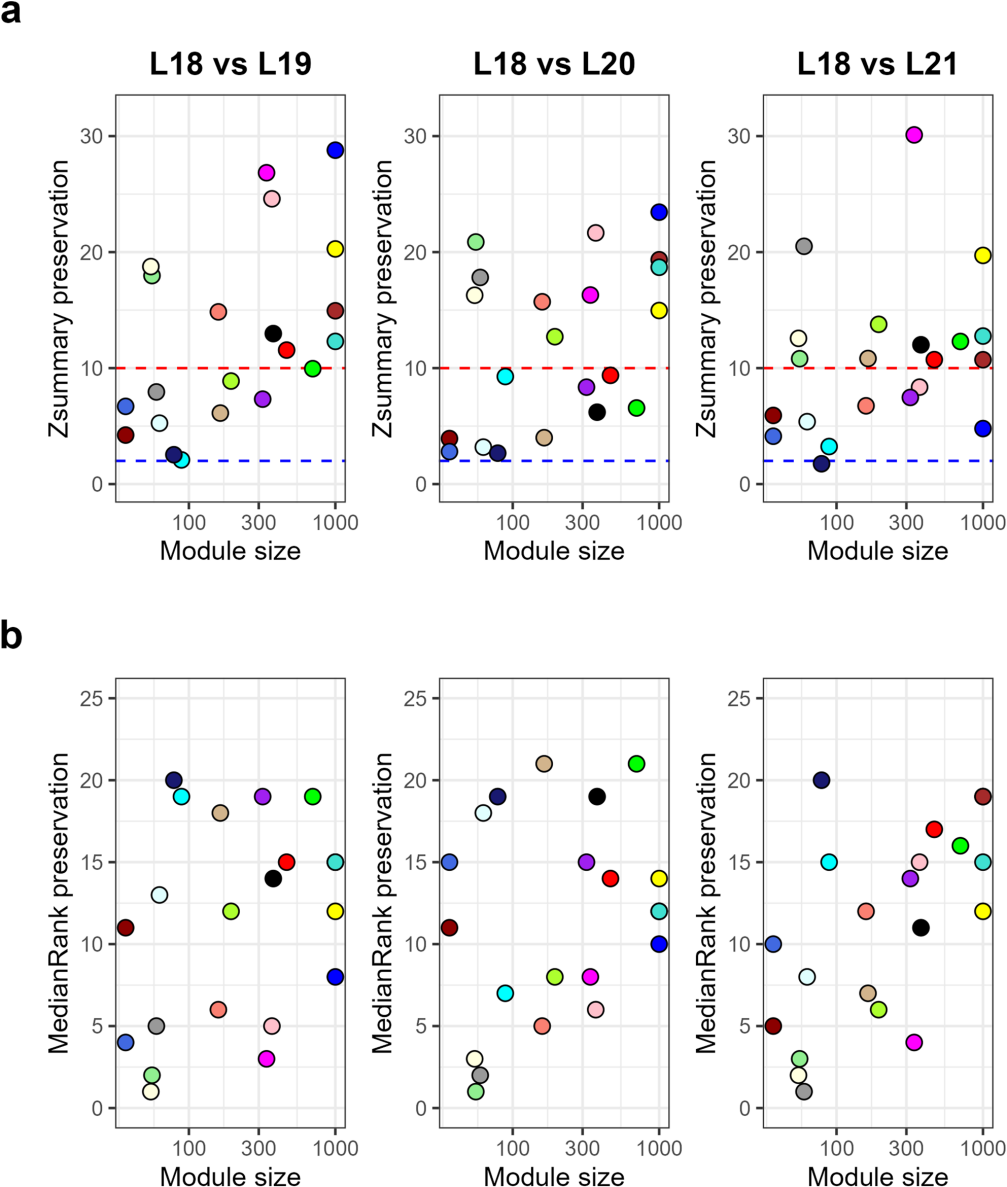
Module preservation across subsequent growing years. Preservation statistics calculated as a function of module size using 2018 as the reference network. Modules are represented by their color. **a** Zsummary statistic, with the red line indicating high preservation (Zsummary > 10) and the blue line indicating no preservation (Zsummary < 2). **b** Median rank statistic

Preservation of the modules from the 2018 network in the other three years of data was calculated using the modulePreservation() function from the WGCNA R package. The 2018 dataset was selected as the reference network for preservation analysis because its network topology, specifically the scale-free topology fit index (R2) and mean connectivity, most closely adhered to the expected curves for a robust biological network [8]. This provided the most mathematically stable baseline against which to test the preservation of modules across the more variable subsequent years. Median rank of the Zsummary was also used to order modules by their preservation summary statistic. These two metrics were plotted as a function of module size as seen in Fig. 4.

The modules significantly correlated to 100-seed weight were tabulated along with their preservation statistics as seen in Table 1. Modules that were highly preserved in at least two of the three other networks (2019 to 2021) when compared to the 2018 network were retained for functional analysis; these modules were “black” (2018 versus 2019 Zsummary score = 12.98, 2018 versus 2020 Zsummary score = 6.20, and 2018 versus 2021 Zsummary score = 12.01), “greenyellow” (2018 versus 2019 Zsummary score = 8.88, 2018 versus 2020 Zsummary score = 12.71, and 2018 versus 2021 Zsummary score = 13.77), “pink” (2018 versus 2019 Zsummary score = 24.60, 2018 versus 2020 Zsummary score = 21.66, and 2018 versus 2021 Zsummary score = 8.36), “red” (2018 versus 2019 Zsummary score = 11.55, 2018 versus 2020 Zsummary score = 9.38, and 2018 versus 2021 Zsummary score = 10.73), and “yellow” (2018 versus 2019 Zsummary score = 20.27, 2018 versus 2020 Zsummary score = 14.96, and 2018 versus 2021 Zsummary score = 19.71).

**Table 1:** Module preservation statistics. Zsummary statistics for modules from the 2018 reference network significantly correlated to 100-seed weight. Bold module assignments indicate strongly preserved modules (Zsummary > 10 in at least two of the three comparative years)

Module Assignment	Zsummary (2018 v 2019)	Zsummary (2018 v 2020)	Zsummary (2018 v 2021)
royalblue	6.70	2.80	4.13
**pink**	24.60	21.66	8.36
cyan	2.09	9.27	3.24
**greenyellow**	8.88	12.71	13.77
green	9.95	6.56	12.30
midnightblue	2.53	2.67	1.75
**yellow**	20.27	14.96	19.71
**black**	12.98	6.20	12.01
**red**	11.55	9.38	10.73
darkred	4.23	3.92	5.92

Gene lists of these five modules were extracted and can be found under Additional file 4. Module “black” contained 377 genes, module “greenyellow” contained 194 genes, module “pink” contained 369 genes, module “red” contained 465 genes, and module “yellow” contained 1072 genes. GO term enrichment analysis was performed per module to determine functions and processes associated with the co-expressing genes using SoyBase’s GO enrichment tool. Overrepresented biological processes with a Bonferroni adjusted p-value of less than *0.01* were used for module associations. Module “black” was enriched for circadian rhythm. Module “greenyellow” was enriched for multiple terms related to photosynthesis (“photosynthesis, light”, “protein-chromophore”, “photosynthesis”, “response to light stimulus”, etc.). Module “pink” was not significantly enriched for broad GO terms, a common phenomenon in highly specific co-expression networks where statistical power is diluted by the high proportion of uncharacterized genes in the soybean genome. However, subsequent hub gene extraction effectively identified its core functional components. Module “red” was enriched for “phosphorelay signal transduction system” and “ergosterol biosynthetic process”. Module “yellow” was enriched for “cell redox homeostasis”.

### Core circadian and lipid metabolism genes drive the preserved networks

Hub genes were extracted from the highly preserved modules using GS and kME measures. Genes with strong expression–trait correlations (|r| ≥ 0.5) and high within-module connectivity (kME ≥ 0.8) were identified as primary nodes in the co-expression networks. The top ten hub genes for each module are summarized in Additional file 5.

Using the annotations in Additional file 5, the hub genes from each module were analyzed for their relevancy to seed weight related pathways. In the “black” module, two MYB transcription factors (*Glyma.18G044200* with a kME of 0.98 and *Glyma.14G210600* 0.97) were identified as homologs of *Arabidopsis Reveille 1*. Both of these genes were annotated with circadian rhythm, photoperiodism, and auxin biosynthetic processes. Additional hub genes included several kinases and phosphatases (*Glyma.13G336300* 0.96, *Glyma.13G211800* 0.95, and *Glyma.05G245300* 0.95), and a lipase (*Glyma.09G041200* 0.95) annotated for lipid catabolic processes.

The “greenyellow” module contained multiple light-harvesting chlorophyll a/b-binding (LHCB and LHCA) proteins (*Glyma.08G180000* with a kME of 0.93, *Glyma.08G074000* 0.92, and *Glyma.15G052400* 0.94), as well as enzymes involved in chlorophyll and heme biosynthesis (*Glyma.14G185700* 0.91). These annotations were consistent with the module’s enrichment for photosynthesis-related terms.

In the “pink” module, hub genes included enzymes related to lipid metabolism (*Glyma.05G163400* with a kME of 0.90 and *Glyma.15G221600* 0.89), cell wall modification (*Glyma.20G141800* 0.94), and signal transduction (*Glyma.11G179600* 0.91 and *Glyma.11G182600* 0.90). Among these, *Glyma.15G221600* encodes *Wrinkled1*, a transcription factor regulating fatty acid biosynthetic processes [30].

The “red” and “yellow” modules contained fewer well-characterized hub genes but were enriched for enzymes involved in primary metabolic pathways. The “red” module included an L-aspartate oxidase (*Glyma.05G007600* with a kME of 0.88), an enzyme in nicotinamide adenine dinucleotide (NAD) and amino acid metabolism [31]. The “yellow” module contained a glucose-1-phosphate adenylyltransferase (*Glyma.06G011700* 0.91), which is a key enzyme in starch biosynthesis [32]. These modules represent metabolic networks associated with energy, carbohydrate, and oil-related processes.

### Network hub genes tightly co-localize with known seed weight QTLs

**Fig. 5:**
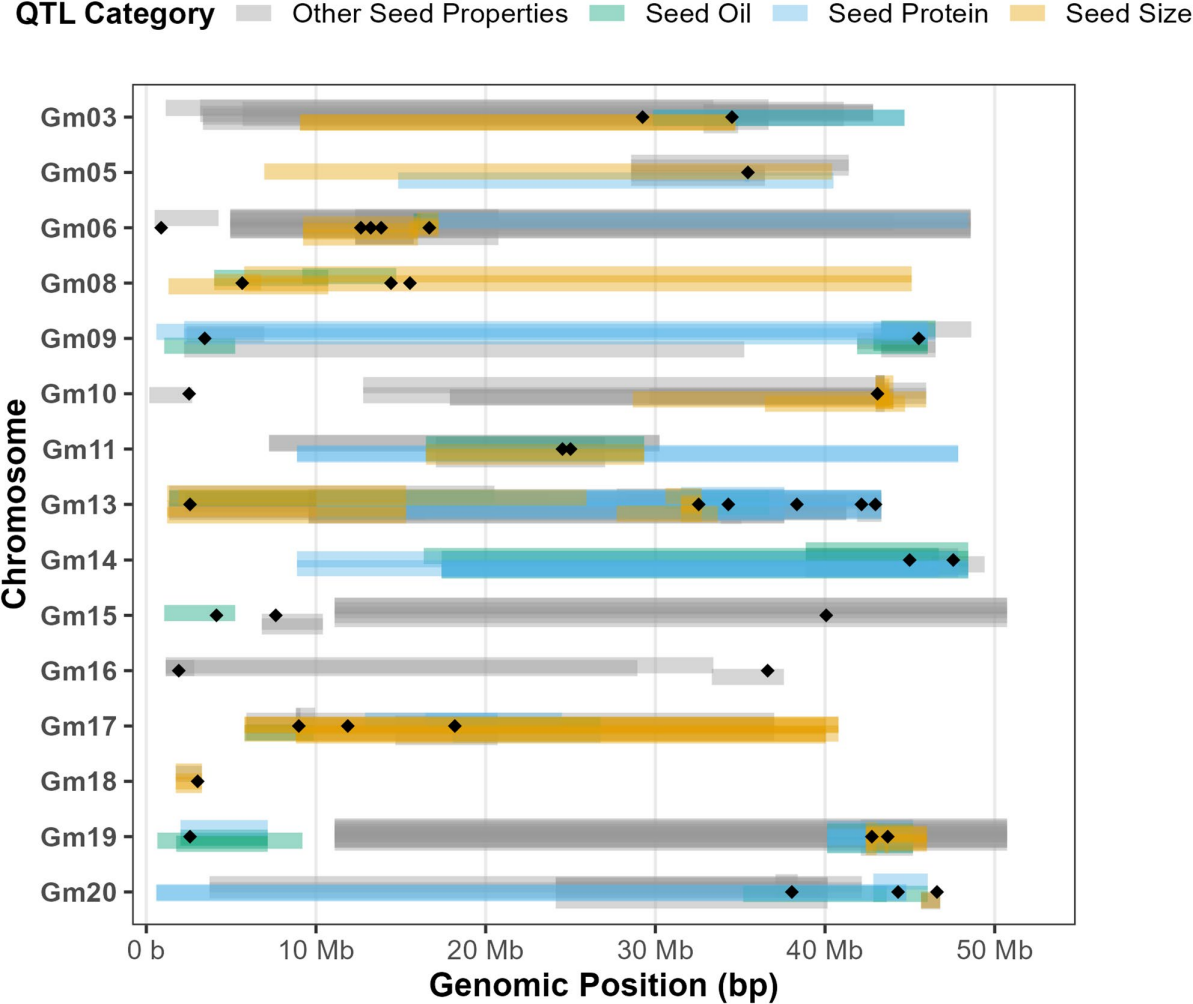
Genomic co-localization of network hub genes with known seed traits. Genomic map of the 40 hub genes associated with seed-related quantitative trait loci (QTLs). Gene locations are indicated by black points, and QTLs are depicted as colored bars extending across their genomic range based on trait association

The top ten hub genes from the five preserved modules associated with 100-seed weight were co-localized with known QTLs for seed traits. 40 of the 50 hub genes were within known seed trait QTLs. The QTLs and the genes associated with them are detailed in Additional file 6. The localization of these genes and their QTLs can be seen in Fig. 5. Of these genes, 18 were found within seed weight QTLs. Chromosomes 6 and 13 had the most genes fall within analyzed QTL regions with five each. The genes on chromosome 6 were primarily in QTLs for seed weight and those on chromosome 13 were in seed protein QTLs. No overlapping regions were found on chromosomes 1, 2, 4, 7, and 12.

### Regional differential expression highlights geographically driven regulatory targets

**Fig. 6:**
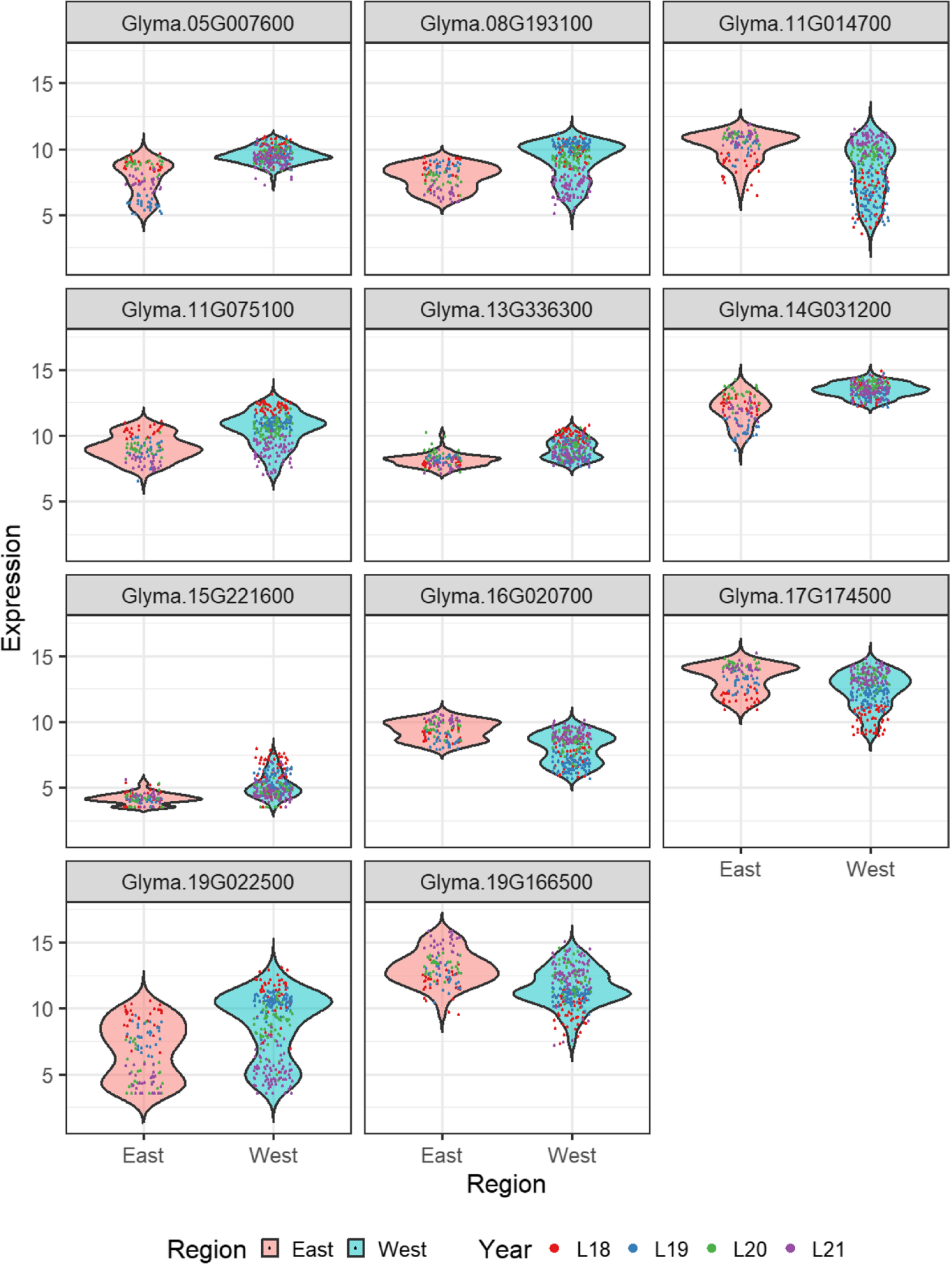
Regional expression differences of candidate hub genes. Expression comparison between samples from Eastern Canada (Ottawa) and Western Canada (Brandon, Morden, Saskatoon) across four years (18 = 2018; 19 = 2019; 20 = 2020; 21 = 2021) for the 11 hub genes exhibiting significant differential expression (FDR-adjusted *p*-value < *0.01*).

To investigate the relationship between hub gene expression and geographic region, the VST expression values of the top 10 hub genes from the five preserved modules were compared between eastern (Ottawa) and western (Brandon, Morden, Saskatoon) locations using the Wilcoxon rank-sum test. P-values were adjusted for multiple testing using the Benjamini–Hochberg procedure. Eleven genes showed consistent differential expression over all years (adjusted p-value < *0.01*) as seen in Table 2. Given the year-to-year variability, we prioritized genes with the strongest statistical evidence. The three hub genes with the most significant FDR-adjusted p-values across the regional comparison (*Glyma.05G007600*, *Glyma.14G031200*, and *Glyma.17G174500*) were identified as the most robust candidates for regional differentiation. *Glyma.05G007600* is an L-aspartate oxidase co-localized with many seed oil content QTLs as determined by the QTL analysis. *Glyma.14G031200* has an *Arabidopsis* closesthomologue annotated as a phytoene synthase. *Glyma.17G174500* is a zeaxanthin epoxidase and is found within a seed weight QTL. An expression plot comparing the two locations demonstrates the yearly variability present in even the statistically significant eleven genes as seen in Fig. 6.

**Table 2:** Differential expression analysis of geographically-driven hub genes. Log fold change values comparing Western to Eastern expression. A positive value indicates upregulated expression in the West, while a negative value indicates downregulated expression in the West

Gene	Year	Log Fold Change	Adjusted *P*-Value
*Glyma.05G007600*	2018	1.17	*6.24E-10*
*Glyma.05G007600*	2019	3.72	*1.33E-15*
*Glyma.05G007600*	2020	0.68	*2.55E-06*
*Glyma.05G007600*	2021	1.81	*5.58E-13*
*Glyma.08G193100*	2018	1.40	*3.95E-09*
*Glyma.08G193100*	2019	1.65	*1.33E-15*
*Glyma.08G193100*	2020	1.57	*1.47E-12*
*Glyma.08G193100*	2021	0.63	*2.51E-03*
*Glyma.11G014700*	2018	-2.16	*2.02E-09*
*Glyma.11G014700*	2019	-3.81	*3.07E-15*
*Glyma.11G014700*	2020	-1.37	*1.39E-12*
*Glyma.11G014700*	2021	-0.41	*7.69E-03*
*Glyma.11G075100*	2018	1.66	*5.01E-13*
*Glyma.11G075100*	2019	1.93	*1.33E-15*
*Glyma.11G075100*	2020	1.52	*5.09E-12*
*Glyma.11G075100*	2021	0.91	*3.60E-06*
*Glyma.13G336300*	2018	2.12	*5.33E-13*
*Glyma.13G336300*	2019	0.14	*1.20E-02*
*Glyma.13G336300*	2020	0.66	*1.79E-04*
*Glyma.13G336300*	2021	0.84	*1.35E-09*
*Glyma.14G031200*	2018	1.31	*2.95E-09*
*Glyma.14G031200*	2019	2.79	*1.33E-15*
*Glyma.14G031200*	2020	0.61	*9.85E-06*
*Glyma.14G031200*	2021	1.35	*4.38E-13*
*Glyma.15G221600*	2018	2.14	*6.46E-10*
*Glyma.15G221600*	2019	1.25	*1.46E-15*
*Glyma.15G221600*	2020	0.44	*2.90E-05*
*Glyma.15G221600*	2021	0.29	*1.64E-03*
*Glyma.16G020700*	2018	-1.38	*1.46E-09*
*Glyma.16G020700*	2019	-1.70	*2.48E-15*
*Glyma.16G020700*	2020	-1.13	*2.26E-10*
*Glyma.16G020700*	2021	-1.12	*4.38E-13*
*Glyma.17G174500*	2018	-1.42	*4.07E-12*
*Glyma.17G174500*	2019	-0.86	*3.38E-08*
*Glyma.17G174500*	2020	-0.94	*6.91E-11*
*Glyma.17G174500*	2021	-0.50	*1.02E-05*
*Glyma.19G022500*	2018	1.70	*1.02E-07*
*Glyma.19G022500*	2019	2.53	*9.94E-15*
*Glyma.19G022500*	2020	3.92	*1.33E-10*
*Glyma.19G022500*	2021	0.83	*9.06E-04*
*Glyma.19G166500*	2018	-1.93	*6.75E-10*
*Glyma.19G166500*	2019	-1.40	*6.98E-12*
*Glyma.19G166500*	2020	-0.98	*4.99E-04*
*Glyma.19G166500*	2021	-2.17	*7.37E-12*

## Discussion

Understanding the genetic basis of soybean seed weight is difficult due to confounding environmental factors that vary by year and location [33]. To overcome this limitation, our study leverages a uniquely robust experimental design, integrating a massive volume of transcriptomic data spanning four distinct growing seasons and two starkly contrasting Canadian mega-environments. Using preservation analyses in combination with co-expression networks across this unprecedented breadth of phenotypic variance helps to effectively eliminate transient environmental noise. The resulting stability of these networks is key to discovering core regulatory mechanisms associated with seed weight that have potential to serve as breeding targets for geographically-tailored soybean lines for Canada’s diverse growing regions. The five identified modules represent distinct biological pathways that underpin seed weight through the source-sink framework [34]. We hypothesized that leaf transcriptional states during the R5 stage, the critical onset of seed filling, act as primary upstream regulators of carbon allocation capacity, ultimately influencing final seed weight. In this context, leaves act as the “source,” synthesizing photoassimilates, while developing seeds act as the “sink,” importing these metabolites for storage.

Modules “black” and “greenyellow” represent the source side of resource acquisition, enriched for circadian rhythm and photosynthesis, respectively. Together, these modules describe the upstream molecular mechanisms that influence carbon assimilation capacity. For example, the “greenyellow” module is driven by light-harvesting chlorophyll a/b-binding (*LHCB*) hub genes. These proteins are fundamental for capturing photon energy to drive the photosynthetic electron transport chain, generating the adenosine triphosphate (ATP) and nicotinamide adenine dinucleotide phosphate (NADPH) required to fix carbon dioxide (CO_2_) into transient starch and transportable sucrose [35]. To optimize this energy capture, genes within the “black” module have critical roles in coordinating the temporal regulation of these processes. The identification of two *Reveille 1* homologs as primary hub genes in this module provides a direct mechanistic link. As master regulators of the circadian clock, the *Reveille* gene family anticipates dawn to prime photosynthetic machinery, regulates daily stomatal conductance to maximize gas exchange, and paces nocturnal starch degradation to ensure a continuous supply of carbon to sink tissues [36]. By optimizing the daily timing and efficiency of carbon fixation, these synchronized pathways directly govern the total pool of photoassimilates available for phloem loading and translocation to the developing seeds, thereby acting as the primary limiting factor for final seed weight.

Modules “pink”, “red”, and “yellow” represent the sink side of resource acquisition with GO terms related to energy storage and primary metabolism pathways, ultimately leading to the development of seeds. Module “pink” contains well-characterized lipid regulators, such as *Glyma.15G221600* a *Wrinkled 1* homologue, governing the conversion of carbon into oil and cuticle components. This gene family is well-characterized as a master controller of seed oil content [30, 37, 38] and is a main contributor to seed filling [39]. The accumulation of oil in the seed is an important determinant of seed weight, and, along with seed protein content, these traits are typically highly correlated [40]. Module “red” contains key machinery for nutrient loading. The identification of a plasma membrane H+-ATPase (*Glyma.06G189900*) suggests this module generates the proton gradient required for active nutrient transport, while starch-degrading enzymes like beta-amylase (*Glyma.15G098100*) imply a role in remobilizing transient carbon reserves for seed filling. Module “yellow” appears to coordinate storage with stress responses, evidenced by the presence of AGPase (*Glyma.06G011700*), the rate-limiting enzyme for starch biosynthesis, alongside stress-signaling genes like zeaxanthin epoxidase (*Glyma.17G174500*). Together, these source and sink modules collaboratively regulate the entire pod-filling pipeline, from *Reveille 1* controlled photosynthetic capture in the leaf, to *Wrinkled 1* directed lipid storage in the developing seeds. This dynamic highlights the species-specific characteristics of soybean seed development. Unlike monocots such as rice, where final seed size is largely physically restricted by the volume of the spikelet hull and less so by the source-sink framework [41, 42], or the model dicot Arabidopsis, which lacks the massive agronomic sink demands of a legume crop, soybean seed weight is uniquely constrained by the immense biochemical energy required for concurrent lipid and protein biosynthesis. Consequently, the collaborative efficiency of this specific source-to-sink transport network acts as the ultimate rate-limiting bottleneck for soybean seed weight.

The identification of major transcription factors in both the source and sink side of energy acquisition and use is indicative of the effectiveness of WGCNA at creating co-expression networks associated with traits. In this case, the presence of both *Reveille 1* and *Wrinkled 1* genes in preserved modules highlights the association of these networks with seed weight through the source sink framework. Interestingly, the master regulators of circadian rhythm (*Reveille 1*) wasnot differentially expressed between Eastern and Western Canada, thus suggesting that the core circadian clock gene acts as a stable, conserved metronome across all environments, maintaining a baseline rhythm for photosynthesis regardless of geography. In contrast, the *Wrinkled 1* gene *Glyma.15G221600* was found to be consistently differentially expressed over the four years with higher expression in the West. While this finding may appear counterintuitive given the significantly lower seed weights observed in the Western samples, it highlights a known phenomenon of transcript level alterations under environmental stress [43, 44]. WRI1 initiates the cascade for carbon-to-oil conversion, but high transcript abundance does not guarantee high terminal metabolite accumulation. To physiologically validate this, we analyzed the corresponding seed oil content from these harvests using a linear mixed model. Despite the upregulation of *Wrinkled 1*, seed oil content was significantly lower in the Western samples (*p* = 1.29e-05). We propose that the consistent upregulation of *Wrinkled 1* in the West is a compensatory transcriptional response. Facing a shorter growing season and geographically-induced photosystem stress, the Western plants upregulate these lipid-regulating transcription factors in an attempt to accelerate seed filling and meet the metabolic demands of the sink tissue. Ultimately, however, the harsh environmental bottlenecks prevent this enhanced transcriptional demand from being physiologically realized, resulting in smaller terminal seed weights and lower oil content. This highlights *Wrinkled 1* not just as a lipid regulator, but as a highly sensitive, data-validated environmental stress indicator in Canadian soybeans.

The importance of these modules as key regulators for seed weight is further reinforced by the co-localization of 18 of the top 50 hub genes with known QTLs for seed weight [24]. As seen in Fig. 5, these QTLs often overlap with those for seed protein content and seed oil content. Combining these findings with differential expression analysis identifies key regulators of geographical adaptation. This convergence of network stability, differential expression, and genetic mapping makes them ideal candidates for breeding strategies aimed at optimizing seed weight for specific latitudes. While the 10 genotypes utilized in this study provide highly robust, agriculturally relevant targets for Canadian breeding programs, they represent a relatively narrow subset of global soybean diversity. Future studies incorporating broader global diversity panels will be crucial to determine if additional, novel regulatory modules govern seed weight in broader germplasm.

It is necessary to acknowledge that R5 leaf tissue was used for this analysis, thus leading to a potential limitation - studying seed traits using leaf expression. While a valid concern, leaves are the source of the carbon required to grow quality seeds. Previous physiological studies have demonstrated a strong causal link between leaf source activity during early seed filling (R5) and final yield components, as the photosynthetic rate and photoassimilate export capacity of the leaf directly limit seed weight [45, 46]. It has also been shown that leaf gene expression influences seed traits through the source-sink framework [9, 47]. Future research could solidify these findings by establishing similar regulatory networks in sink tissue during the same developmental stage. Understanding the biochemical pathways involved in the seed storage of this carbon is imperative to discovering regulatory networks associated with this process. These five modules emphasize the connection between leaf gene expression and seed weight through pathways involving photosynthesis, circadian rhythm, and metabolism.

To translate these findings into practical agronomic applications, the identified hub genes serve as high-confidence targets for future allele mining. While the differential expression of genes like *Wrinkled 1* demonstrated environmental plasticity in this adapted Canadian germplasm, screening diverse global soybean accessions may reveal novel structural or promoter alleles that confer more robust lipid accumulation under short-season stress. Once favorable allelic variants of these core network genes are identified in unadapted germplasm, high-throughput molecular markers (such as Kompetitive Allele Specific PCR (KASP) assays) can be designed to introgress these targeted stress-tolerance and phenological alleles into elite Canadian breeding lines.

## Conclusions

This study demonstrated that WGCNA is an effective tool for discerning stable genetic regulators of soybean seed weight while controlling for yearly environmental variability. By using module preservation analysis across four growing years of data, five robust modules significantly associated with seed weight were identified. These modules were supported by the finding of *Reveille 1* and *Wrinkled 1* transcription factors in the hub genes along with others that demonstrated the importance of the source-sink framework for this trait. Co-localization of these hub genes with QTLs for seed weight further solidified the network analysis and provided strong support for these genes as candidates for marker-assisted selection. Combining this analysis with the differential expression analysis allowed for the identification of key seed weight regulators that are geographically-driven, thus providing a more targeted set of genes for soybean breeders. Finally, distinguishing between stable regulators (*Reveille 1*) and plastic regulators (*Wrinkled 1*) provides a targeted set of genes for breeders to fine-tune soybean varieties for specific geographic regions.

## Supplementary Information

Supplementary Material 1. Phenotypic data. Data gathered for each harvest from 2018 to 2021 including sample names and agronomic traits.

Supplementary Material 2. Weather data. Data gathered for each station over the course of the experiment (2018 to 2021) for weather and climate.

Supplementary Material 3. Raw count matrix. Raw counts of RNA sequences from all samples (2018 to 2021).

Supplementary Material 4. Gene assignments. Module assignments of the genes found in the five modules mentioned in the text.

Supplementary Material 5. Hub genes. Top ten genes with annotations for each of the five modules of interest by highest intramodular connectivity.

Supplementary Material 6. QTL associations. The 40 seed trait related QTLs and the genes that fall within their regions.

## Data Availability

All data generated or analysed during this study are included in this published article (and its additional files).

## Abbreviations

BP: Biological Process
DE: Differential Expression
FDR: False Discovery Rate
GO: Gene Ontology
GS: Gene Significance
kME: Module Membership
ME: Module Eigengene
MF: Molecular Function
QTL: Quantitative Trait Locus
VST: Variance Stabilizing Transformation
WGCNA: Weighted Gene Co-expression Network Analysis
